# Comparison of the safety and effectiveness of the four-hook needle and hook wire for the preoperative positioning of localization ground glass nodules

**DOI:** 10.1186/s13019-024-02497-1

**Published:** 2024-01-31

**Authors:** Yongming Wang, Lijun Jing, Changsheng Liang, Junzhong Liu, Shubo Wang, Gongchao Wang

**Affiliations:** 1 Department of Thoracic Surgery, Shandong Provincial Hospital, Shandong University, Jinan, 250021 Shandong China; 2 Department of Thoracic Surgery, Weifang No.2 Peoplès Hospital, Weifang, 261041 Shandong China; 3 Department of Anesthesiology, Weifang No.2 Peoplès Hospital, Weifang, 261041 Shandong China; 4 Department of Radiology, Weifang No.2 Peoplès Hospital, Weifang, 261041 Shandong China

**Keywords:** Hook wire, Ground glass nodule, Localization, Thoracic surgery

## Abstract

**Background:**

With the implementation of lung cancer screening programs, an increasing number of pulmonary nodules have been detected.Video-assisted thoracoscopic surgery (VATS) could provide adequate tissue specimens for pathological analysis, and has few postoperative complications.However, locating the nodules intraoperatively by palpation can be difficult for thoracic surgeons. The preoperative pulmonary nodule localization technique is a very effective method.We compared the safety and effectiveness of two methods for the preoperative localization of pulmonary ground glass nodules.

**Methods:**

From October 2020 to April 2021, 133 patients who underwent CT-guided single pulmonary nodule localization were retrospectively reviewed. All patients underwent video-assisted thoracoscopic surgery (VATS) after successful localization. Statistical analysis was used to evaluate the localization accuracy, safety, information related to surgery and postoperative pathology information. The aim of this study was to evaluate the clinical effects of the two localization needles.

**Results:**

The mean maximal transverse nodule diameters in the four-hook needle and hook wire groups were 8.97 ± 3.85 mm and 9.00 ± 3.19 mm, respectively (*P* = 0.967). The localization times in the four-hook needle and hook wire groups were 20.58 ± 2.65 min and 21.43 ± 3.06 min, respectively (*P* = 0.09). The dislodgement rate was significantly higher in the hook wire group than in the four-hook needle group (7.46% vs. 0, *P* = 0.024). The mean patient pain scores based on the visual analog scale in the four-hook needle and hook wire groups were 2.87 ± 0.67 and 6.10 ± 2.39, respectively (*P* = 0.000). All ground glass nodules (GGNs) were successfully resected by VATS.

**Conclusions:**

Preoperative pulmonary nodule localization with both a four-hook needle and hook wire is safe, convenient and effective.

## Background

Lung cancer is the leading cause of cancer-related deaths [1]. Although the 5-year survival rate of lung cancer is only 15%, early diagnosis and treatment can increase it to 50% [2]. With the widespread use of low-dose spiral computed tomography (CT), an increasing number of pulmonary nodules can be detected [3]. These pulmonary nodules take on three forms on CT: pure ground glass nodules (GGNs), mixed GGNs, and solid nodules. Studies have shown that the malignant percentage of pure GGNs is 59–73%, whereas that of pure solid nodules is 7–9% [4]. Therefore, nodules that are classified based on the relevant imaging manifestations should be subjected to further pathological evaluation, whether by percutaneous lung biopsy, bronchoscopy or minimally invasive thoracoscopic surgery [5]. Some nodules are smaller in diameter, have fewer solid components, or are located deep in the lungs. Routine aspiration biopsy or needle aspiration biopsy for pathology becomes a challenge. Some scholars report that the accuracy of puncture biopsy for less than 1 cm lung nodules under CT guidance is only 48.5% [6]. When nonsurgical biopsy is not available or if the diagnosis is unclear, thoracic surgical biopsy is needed to further clarify the pathological diagnosis. Compared with conventional thoracotomy, thoracoscopy has advantages because it does not require conventional thoracotomy and causes less trauma [7]. Video-assisted thoracoscopic surgery (VATS) provides adequate tissue specimens for pathological analysis, is more comfortable and has fewer postoperative complications, making it ideal for the treatment of pulmonary nodules. Compared with thoracotomy, VATS not only provides adequate tissue specimens for pathological analysis but is also more comfortable, which makes it an ideal treatment for pulmonary nodules [7, 8]. Due to the small pulmonary nodules, deep location or soft nodule texture, it is difficult to manually reach nodules with just the fingers during VATS, and this can even lead to the need for conventional thoracotomy surgery. It may even lead to a routine thoracotomy. The preoperative pulmonary nodule localization technique is a very effective method to mitigate this challenge. Currently, a variety of auxiliary devices can be used, including hook wire, protein glue localization, methylene blue injection, intraoperative ultrasound localization, intraoperative electromagnetic navigation, and immunofluorescence methods [9–14]. Each method has its own advantages and disadvantages. Among these methods, the hook wire was one of the first applied lung nodule needles and is very popular in positioning, with a success rate of up to 96%. The four-hook needle is a modified localization needle for pulmonary nodules. Currently, few articles have compared the two methods. The purpose of this study was to compare the safety and effectiveness of the two mentals for the preoperative localization of pulmonary nodules.

## Methods

### Materials

We retrospectively analyzed the clinical data of 180 consecutive patients who underwent preoperative localization in the Department of Thoracic Surgery of our institution from October 2020 to April 2021. All patients underwent routine pulmonary function examination, craniocerebral magnetic resonance imaging or CT, systematic bone scan, abdominal ultrasound or CT to exclude possible metastases, and analysis of related tumor markers. After plain or enhanced CT scans, all images were transferred to an AW4.6 workstation (GE Healthcare, Chicago, IL, USA) for postprocessing. The inclusion criteria for VATS for patients with GGN were as follows: [1] GGN confirmed by high-resolution CT; [2] single GGN; [3] GGN difficult to detect during an operation; [4] GGN suspected of being malignant; and [5] distance from the pleura of 5-40 mm. The exclusion criteria were as follows: multiple lesions and the identification of two or more GGNs simultaneously. There were 133 eligible patients.

### CT-guided localization process

**Fig. 1:**
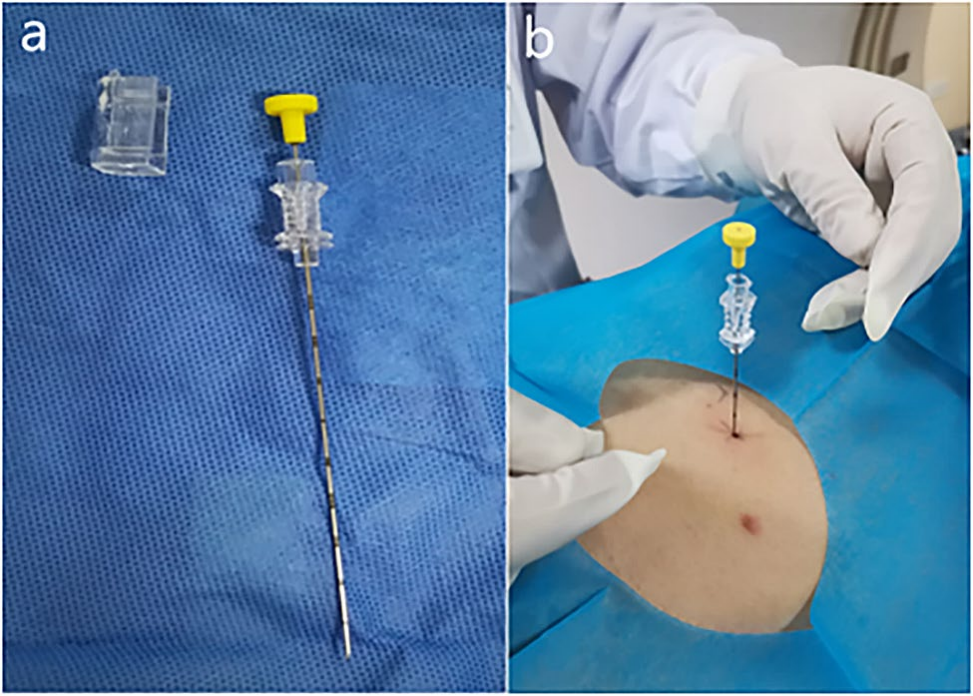
(**a**) Picture of the entire localization needle. (**b**) Preoperative positioning

**Fig. 2:**
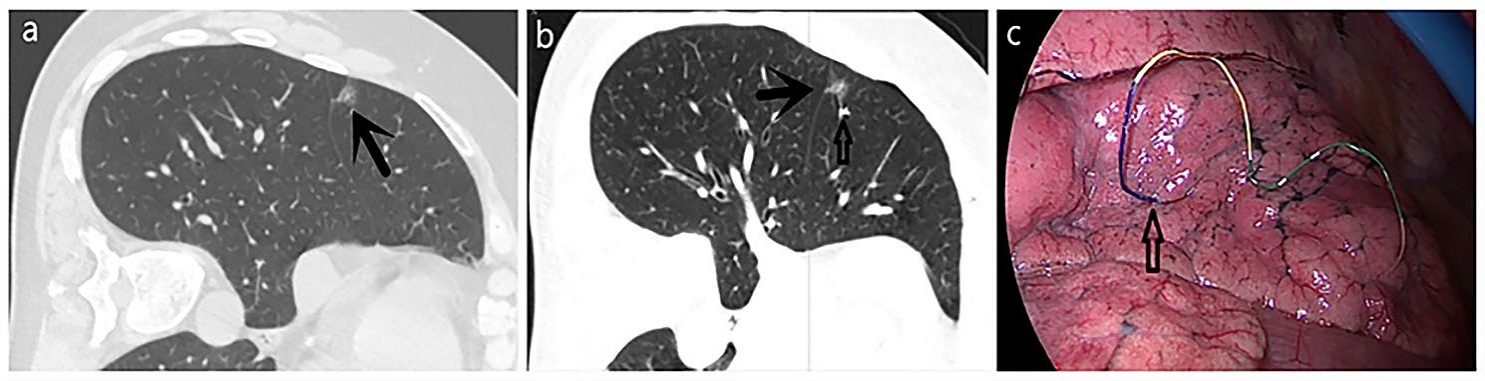
A 56-year-old female with a GGN in the right middle lobe who underwent CT-guided localization with a four-hook needle. (**a**) The lesion (black arrow) is observed on an axial CT image. (**b**) Postlocalization CT shows the relationship among the released anchor (white arrow), lesion (black arrow) and chest wall. (**c**) The end of the four-hook needle (white hollow arrow) is visualized during VATS.

**Fig. 3:**
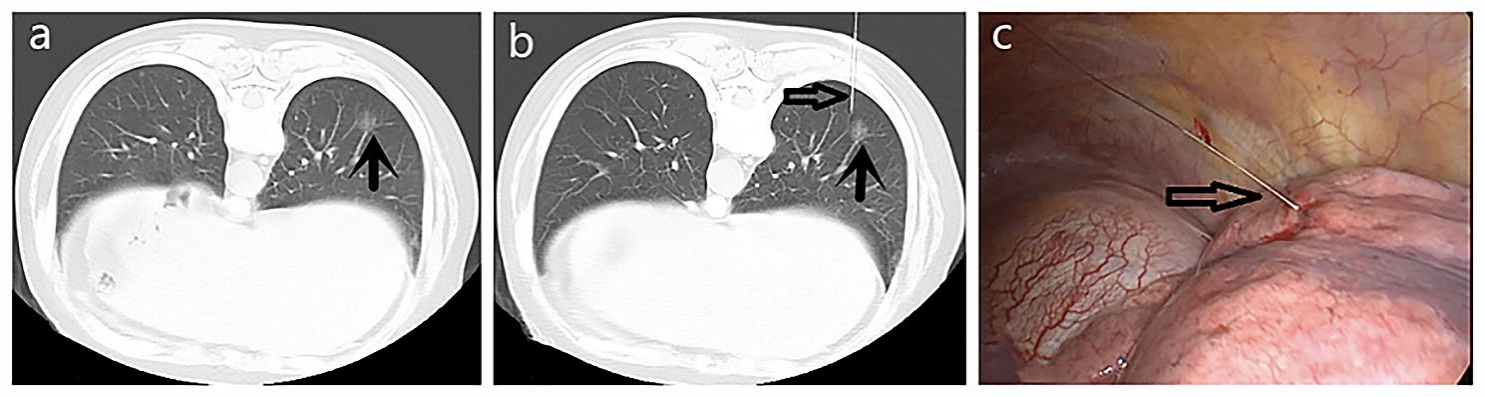
A 65-year-old female with a GGN in the right lower lobe who underwent CT-guided localization with a hook wire needle. (**a**) The lesion (black arrow) is observed on an axial CT image. (**b**) A hook wire needle (black arrow) is inserted around the lesion; postlocalization CT shows the relationship among the needle (black arrow), lesion (white arrow) and chest wall. (**c**) The end of the four-hook needle (white hollow arrow) is visualized during VATS.

All CT-guided localizations of GGNs were performed by the same radiologist who had engaged in CT-guided intervention for 15 years. All CT scans were performed on a 64-row multislice spiral CT (GE Healthcare, USA). A four-hook needle (model: SS510-10 Senscure, China) (Fig. 1a and b) or hook wire (20 g×120 mm; Pajunk, Germany) was used for puncture. The four-hook needle is composed of a 19G coaxial needle, tri-colored suture, 4-hook anchor, pusher, release buckle, and protection tube. Before positioning, the surgeon and the radiologist reviewed the CT images together to determine whether the pulmonary nodules could be located. Then, a path that avoided the vessels, trachea and interleaf pleura was determined by the radiologist. After routine disinfection and drape laying, 2% lidocaine was used to locally anesthetize the layers of the chest wall. The localization needle was inserted into the desired depth according to the standard four-hook wire procedure (Fig. 2a) [15].CT was performed again, and the needle inlet angle and depth were adjusted according to the image until the tip was located in the GGN or within 1 cm. The claw hook was released through a puncture needle. After successful localization, another CT scan was performed again to determine the spatial location relationship between the pulmonary nodules and the localization needle (Fig. 3a), as well as if there was pneumothorax and hemorrhage. The processes of hook wire localization were similar to those in the four-hook needle group. After the target nodule was confirmed, a reasonable path was chosen based on the CT images, and then the localization needle was inserted around the nodule (Fig. 3a and b). After successful positioning, the patient was placed in a moving bed or wheelchair and delivered to the operating room.

### Thoracoscopic surgical procedure

After positioning, the patients were immediately transferred to the operating room for thoracoscopic surgery. After successful general anesthesia and endotracheal intubation, the patients were placed in an appropriate lateral decubital position. During thoracoscopic surgery, surgeons can easily determine the location of the pulmonary nodules and anchor depth according to the location of the localization needle (Figs. 2c and 3c). Partial lobe resection (wedge or segment resection) was performed using a margin of more than 2 cm from the edge of the lesion according to the preoperative surgical plan. The resected specimen was subjected to intraoperative rapid frozen pathology examination. The patients with malignant nodules greater than 2 cm in diameter underwent routine lobectomy and lymph node dissection or sampling.All patients received postoperative analgesia with patient controlled intravenous analgesia(PCIA )with sufentanil 0.05ug/Kg.h.

### Variables

The primary outcomes were the marking time and procedure success. The time from obtaining the first CT image to obtaining the last CT image was defined as the marking time. Localization success was defined as a localization marker in or within 10 mm of the nodules measured on the final images of the procedure.The time from cutting the skin to closing the incision was defined as the operative time. Extubation time was the time from pleural drainage tube insertion to removal.Complications (e.g., pneumothorax and pulmonary hemorrhage) were assessed. The pain visual analog scale (0–10) was used to assess the pain level after positioning.

### Statistical analysis

The statistical analysis software that was used was SPSS v21.0 (SPSS Inc., USA). Descriptive statistics for continuous variables are presented as the mean ± standard deviation or median. Continuous variables were analyzed by t tests, and categorical variables were compared by chi-square, Pearson, and Fisher tests. A value of *P* < 0.05 was considered significant.

## Results

In total, 133 patients were included in this study. The clinical characteristics of the patients and nodules are shown in Table 1. There were no significant differences in age, sex, nodule distribution, operative technique or type of nodule between the two groups. The mean maximal transverse nodule diameters in the four-hook needle and hook wire groups were 8.97 ± 3.85 mm and 9.00 ± 3.19 mm, respectively (*P* = 0.967). The mean distances from the nodule margins to the superficial pleura in the four-hook needle and hook wire groups were 10.94 ± 1.63 mm and 11.32 ± 1.36 mm, respectively (*P* = 0.150). The localization times in the four-hook needle and hook wire groups were 20.58 ± 2.65 min and 21.43 ± 3.06 min, respectively (*P* = 0.09). Pneumothorax, hemorrhage and dislodgement were the primary localization complications. In this study, there was no significant difference between the groups regarding pneumothorax or hemorrhage. The dislodgement rate was significantly higher in the hook wire group than in the four-hook needle group (7.46% vs. 0, *P =* 0.024). The mean patient pain scores based on the visual analog scale in the four-hook needle and hook wire groups were 2.87 ± 0.67 and 6.10 ± 2.39, respectively (*P* = 0.000) (Table 2).

**Table 1:** Clinical patient characteristics

	Four-hook needle group (*n* = 66)	Hook wire group (*n* = 67)	t/χ2	*P* value
Age (years)	56.45 ± 11.15	57.24 ± 12.12	-0.388	0.698
Sex			0.678	0.410
Male	23 (33.8%)	28 (41.8%)		
Female	43 (63.2%)	39 (58.2%)		
Size (mm)	8.97 ± 3.85	9.00 ± 3.19	-0.042	0.967
Depth (mm)	10.17 ± 1.99	10.54 ± 1.17	-1.322	0.189
Location			0.675	0.954
Right upper lobe	21 (31.8%)	23 (34.3%)		
Right middle lobe	3 (4.5%)	3 (4.5%)		
Right lower lobe	13 (19.7%)	11 (16.4%)		
Left upper lobe	17 (25.8%)	20 (29.9%)		
Left lower lobe	12 (18.2%)	10 (14.9%)		
Operative technique			1.826	0.401
Wedge resection	51 (77.3%)	56 (83.6%)		
Segmentectomy	7 (10.6%)	3 (4.5%)		
Lobectomy	8 (12.1%)	8 (11.9%)		
Type of nodule			1.141	0.286
Pure GGN	56 (84.8%)	52 (77.6%)		
Mixed GGN	10 (15.2%)	15 (22.4%)		
Operation time (min)	51.29 ± 19.40	47.54 ± 15.89	1.220	0.224
Bleeding volume (ml)	25.23 ± 11.38	23.95 ± 9.98	0.685	0.494
Drainage duration (min)	3.11 ± 0.56	3.10 ± 0.55	0.016	0.987
Hospital stay (days)	6.57 ± 0.79	6.56 ± 0.80	0.062	0.950

GGN: ground-glass nodule

**Table 2:** Localization results

	Four-hook needle group (*n* = 66)	Hook wire group (*n* = 67)	t/χ2	*P* value
Positioning time (min)	20.58 ± 2.65	21.43 ± 3.06	-1.703	0.09
Procedure success	66(100%)	66(98.5%)	0.993	0.319
Pneumothorax	17(25.8%)	19(28.4%)	0.153	0.696
Hemorrhage	9(13.6%)	10(14.9%)	0.045	0.832
Dislodgement	0	5(7.46%)	5.118	0.024
VAS score	2.87 ± 0.67	6.10 ± 2.39	-10.566	0.000

VAS: visual analog scale

The pathological diagnostic results for the GGNs were as follows: invasive adenocarcinoma (*n* = 48), minimally invasive adenocarcinoma (*n* = 56), carcinoma in situ (*n* = 11), atypical adenomatous hyperplasia (*n* = 9), fibroinflammatory nodule (*n* = 5), inflammation (*n* = 2), hamartoma (*n* = 1) and cryptococcus infection (*n* = 1) (Table 3).

**Table 3:** Postoperative pathological results for 133 GGNs

	Number of nodules (*n* = 133)
Invasive adenocarcinoma	48
Minimally invasive adenocarcinoma	56
Carcinoma in situ	11
Atypical adenomatous hyperplasia	9
Fibroinflammatory nodule	5
Inflammation	2
Hamartoma	1
Cryptococcus infection	1

## Discussion

With the implementation of lung cancer screening programs, an increasing number of pulmonary nodules have been detected, which provides a better opportunity for the early diagnosis and treatment of lung cancer. Early surgical treatment will improve the 5-year survival rate of postoperative lung cancer. The precise intraoperative localization of pulmonary nodules is a challenge for every thoracic surgeon. When the nodules are small or are pure ground glass nodules, it is difficult to accurately locate them through observation and touch during the operation, which increases the difficulty of the operation, extends the operation time, and increases the risk of resection of normal lung tissue. Thus, accurate and safe preoperative localization is very important. Previous localization methods have corresponding disadvantages [16]. For example, methylene blue injection may stain widely with dose and time, resulting in inaccurate localization [17]. The safety trauma of spring coil localization is small, but the possibility of shedding is large. In recent years, a new four-hook localization needle has emerged, and the four-hook needle and hook wire methods have become two commonly used localization methods. We retrospectively compared the use of the two localization needles in pulmonary nodule localization. The localization success rates of the four-hook needle and hook wire groups were 100% and 98.5%, respectively. There was no significant difference between the two groups for the marking time.

The hook wire is a commonly used localization tool in recent years and is used initially for breast nodule localization. The front end of the hook wire is a single barb with a rigid tail wire. After positioning, the metal barbs can fix it in the lung tissue. The attached guide wire extends outward across the chest wall and sticks to the skin, so changes in the body position and respiratory movement could lead to the dislodgement or migration of the localization needle and result in localization failure. In this study, dislodgement or migration occurred in 5 patents in the hook wire group, similar to reports in the literature [18]. Location needle decoupling often occurred while the patients were transported to the operating room or while waiting for surgery. Therefore, our experience revealed that after successful positioning, the patient should be transferred immediately to the operating room for surgery. Even if decoupling occurs, the location of the pulmonary nodules can be determined by the hematoma on the surface of the lung during surgery, and the pulmonary nodules can be successfully removed. In this study, the pulmonary nodules were successfully removed in all five decoupled patients in the hook wire group.

The four-hook needle is basically the same as the hook wire. However, the tip claw is composed of 4 metal hook claws, which have a strong binding force with the surrounding tissue, and the position is relatively fixed. The flexible wire left in the chest wall has a weak pull force on the anchor hook, which is not easy to shift while the patients are waiting for surgery, and even if the shallow lesions are located, the anchor hook does not easily fall off and shift. Because the anchoring force of the anchor hook on the surrounding lung tissue is strong enough, the lung tissue in the focal area can be raised through the lifting tricolour line during the operation, and the operative field is exposed easily. In this study, no cases of decoupling or displacement occurred in the four-hook needle group. From our experience at our center, the operator can obviously touch the anchor hook of the anterior segment of the four-hook needle, and the operator can easily determine the nodule position by judging the localization point and anchor hook position.

Pneumothorax and hemorrhage are common complications during localization. In this study, there were no significant differences between the two groups, indicating that the two positioning methods were both effective and safe.None of the patients required special treatment.

After hook wire positioning, the hard wire that was placed through the partial pleura and the chest wall was fixed on the body surface, leading to hard wire rubbing against the wall pleural membrane due to the motion of the lung tissue, resulting in poor experience and pain for the patient [19]. The localization line of the four-hook needle posterior connection is a soft line that can be released into the chest cavity during positioning, which can reduce the stimulation of the mural pleura and significantly reduce the patient’s chest pain [20]. In this study, the average patient pain score was significantly higher in the hook wire group than in the four-hook needle group before VATS resection. According to the standard visual analog scale, the mean patient pain score in the four-hook needle group was 2.87 ± 0.67 in this study, which was similar to the report of 2.96 ± 1.43. Due to the good comfort of the four-hook needle, it was not necessary to perform the surgery immediately after applying the four-hook needle positioning, which made it easier to arrange the localization and operation time [21]. In the hook wire group, one patient had a severe pleural reaction due to pain after localization because after positioning, the patient immediately had chest tightness, dyspnea, a pale face, sweating, and decreased heart rate and blood pressure. The patient’s pleural response symptoms gradually improved after emergency oxygen, fluid rehydration and anti-shock treatment. Some scholars believe that the pleura is rich in sensory nerves, and pain stimulation can lead to an increased incidence of pleural response. Pleural pain caused by puncture localization can stimulate the cerebral cortex and hypothalamus, causing dilation of blood vessels, lowering of blood pressure, slowing heart rate, and even shock in severe cases 22.

Our results have several shortcomings.This study is a single-center retrospective study and it lacks large multicenter, randomized controlled trials to support the present findings. In addition, We did not compare postoperative follow-up for the two localization methods.

## Conclusions

This study identified that the two preoperative localization methods were safe, convenient and effective. There was no difference in the localization time, success rate, pneumothorax or hemorrhage complications. The postoperative localization pain score and dislodgement rate in the four-hook needle group were slightly lower than those in the hook wire group.

## Data Availability

All data generated or analyzed during this study are included in this published article.

## Abbreviations

GGO: Ground-glass opacity
VATS: Video-assisted thoracoscopic surgery
GGN: Ground-glass nodule
